# What percentage of patients with cancer develop hiccups with oxaliplatin- or cisplatin-based chemotherapy? a compilation of patient-reported outcomes

**DOI:** 10.1371/journal.pone.0280947

**Published:** 2023-01-27

**Authors:** Christopher Ehret, Nichole A. Martin, Aminah Jatoi

**Affiliations:** Department of Oncology, Mayo Clinic, Rochester, Minnesota, United States of America; Houston Methodist, UNITED STATES

## Abstract

**Background:**

Chemotherapy-induced hiccups are understudied but can cause sleep deprivation, fatigue, pain in the chest and abdomen, poor oral intake, aspiration, and even death. As a critical next step toward investigating better palliative methods, this study reported patient-reported incidence of hiccups after oxaliplatin- or cisplatin-based chemotherapy.

**Methods:**

The current study relied on 2 previous studies that sought to acquire consecutive direct patient report of hiccups among patients who had recently received chemotherapy with cisplatin or oxaliplatin. These patient-reported data in conjunction with information from the medical record are the focus of this report.

**Results:**

Of 541 patients, 337 were successful contacted by phone; and 95 (28%; 95% CI: 23%, 33%) of these contacted patients reported hiccups. In univariable analyses, male gender (odds ratio (OR): 2.17 (95% confidence ratio (95% CI): 1.30, 3.62); p = 0.002), increased height (OR: 1.03 (95% CI: 1.00, 1.06); p = 0.02), and concomitant aprepitant/fosaprepitant (OR: 2.23 (95% CI: 1.31, 3.78); p = 0.002) were associated with hiccups. In multivariable analyses, these statistically significant associations persisted except for height.

**Conclusions:**

These patient-reported data demonstrate that oxaliplatin- or cisplatin-induced hiccups occur in a notable proportion of patients with cancer. Male gender and concomitant aprepitant/fosaprepitant appear to increase risk.

## Introduction

Chemotherapy-induced hiccups are understudied. However, in some patients with cancer, hiccups spawn sleep deprivation, fatigue, pain in the chest and abdomen, poor oral intake, aspiration, and even death [1]. Certain cancer chemotherapy agents, such as cisplatin and oxaliplatin, either with or without dexamethasone, can cause hiccups [1]. The cyclical nature of hiccups, which, in effect, occur with the administration of each cycle of cancer chemotherapy, can conceivably generate a cumulative burden of hiccups and hiccup-associated symptoms [2]. As a result, cancer patients—who are contending with multiple other symptoms—are also having to endure hiccups and their consequences.

What percentage of cancer patients who receive oxaliplatin- or cisplatin-based chemotherapy experience hiccups? Previous estimates cite 15–40% of patients who receive chemotherapy develop hiccups, but this percentage might be inaccurate for at least three reasons [3, 4]. First, such percentages have at times been derived from databases. Databases require healthcare providers or researchers to ask about hiccups, to have patients recall and report their hiccups, and then to have the healthcare provider or researcher input these responses [5]. Because this is a multi-step process, hiccups might never be recorded in the database. Second, to our knowledge, few studies have assessed hiccup incidence as a patient-reported outcome, or as a sign/symptom that patients directly report. Patient-reported outcomes have garnered more attention of late because they capture symptoms at a higher rate than those that healthcare providers report on behalf of patients, an observation that suggests patient-reported outcomes yield greater accuracy [6]. Finally, to our knowledge, few studies have undertaken consecutive patient recruitment to assess hiccups. Attempting to ask each cancer patient at high-risk for hiccups about this sign/symptom complex would also help ensure accuracy in the reporting of the percentage of patients with hiccups. Although prior studies on hiccup incidence have been of value, a void remains in understanding the true percentage of patients who have cancer and who experience this sign/symptom complex.

The current study was undertaken to fill this void. This study relied on a compilation of data from previous studies of cancer patients at high risk for hiccups; these patients were high risk because they had recently received oxaliplatin- or cisplatin-based chemotherapy. The goal of the current study was to provide accurate benchmark data on the percentage of cancer patients who developed hiccups after such chemotherapy and to thereby foster further research on hiccup palliation.

## Methods

### Overview

The Mayo Clinic Institutional Review Board (IRB) approved this study (#22–001647), which relied on a compilation of data from two previously-conducted parent studies–a qualitative, interview study that explored the burden of hiccups in patients with cancer (#21–008466) and a clinical trial that assessed the feasibility of testing educational materials for hiccup palliation (NCT05313685). Each parent study used the large, multi-site Mayo Clinic clinical volumes for patient recruitment. For the current study, the Mayo Clinic IRB waived patient consent.

### Patient recruitment

For each parent study, the multi-site Mayo Clinic electronic medical record was searched in a real-time manner for eligible patients who 1) were ≥ 18 years old, 2) had a cancer diagnosis, and 3) had ongoing chemotherapy with cisplatin or oxaliplatin chemotherapy. A list of these patients was generated at the beginning of every workday for each of these studies, which sequentially recruited patients, with completion of one study prior to the initiation of the other. For recruitment, patients had been consecutively contacted by phone. An investigator called each patient a few days after receipt of chemotherapy from a daily generated list.

A telephone script was used to assess eligibility and to recruit to the parent studies. This script included the following face-validated statement and question: “Our study focuses only on patients who have recently had hiccups. Have you had hiccups within the last [few] weeks?”

### Data acquisition

Patient responses to the foregoing question were categorized as affirmative for hiccups or not. In some instances, patients were unable to be reached for study recruitment; or, upon phone contact, they declined to answer questions. These patients were included in the dataset for this study but were denoted as not having provided data on hiccups. When possible, the medical records of all patients–regardless of whether phone contact was made and regardless of whether any data on hiccups were available—were reviewed in detail for demographics, confirmation of chemotherapy administration, and the use of other concomitant drugs. Of note, if a patient had been contacted for participation in both studies, the affirmative response to hiccups was included only once to generate independent, nonduplicative data for each patient.

### Reporting and analyses

The primary endpoint of the current study is the percentage of patients who reported hiccups; this percentage is reported along with a 95% confidence interval (CI). A sensitivity analysis was performed to examine whether statistically significant demographic differences were detected among patients with and without data on hiccups. Among patients with data on hiccups, logistic regression models were used to explore literature-based variables of interest and their association with hiccups [4, 5, 7]. To ensure stability of the logistic regression models and to thereby lessen the risk of reporting spurious findings, only a limited number of plausible variables were examined based on the number of patients who reported hiccups; this approach is in keeping with a sound statistical design [8, 9]. Statistically significant associations (p<0.05) derived from the univariable model were included in the multivariable model. Odds ratios along with 95% CI’s are reported.

## Results

### Recruitment and demographics

**Fig 1:**
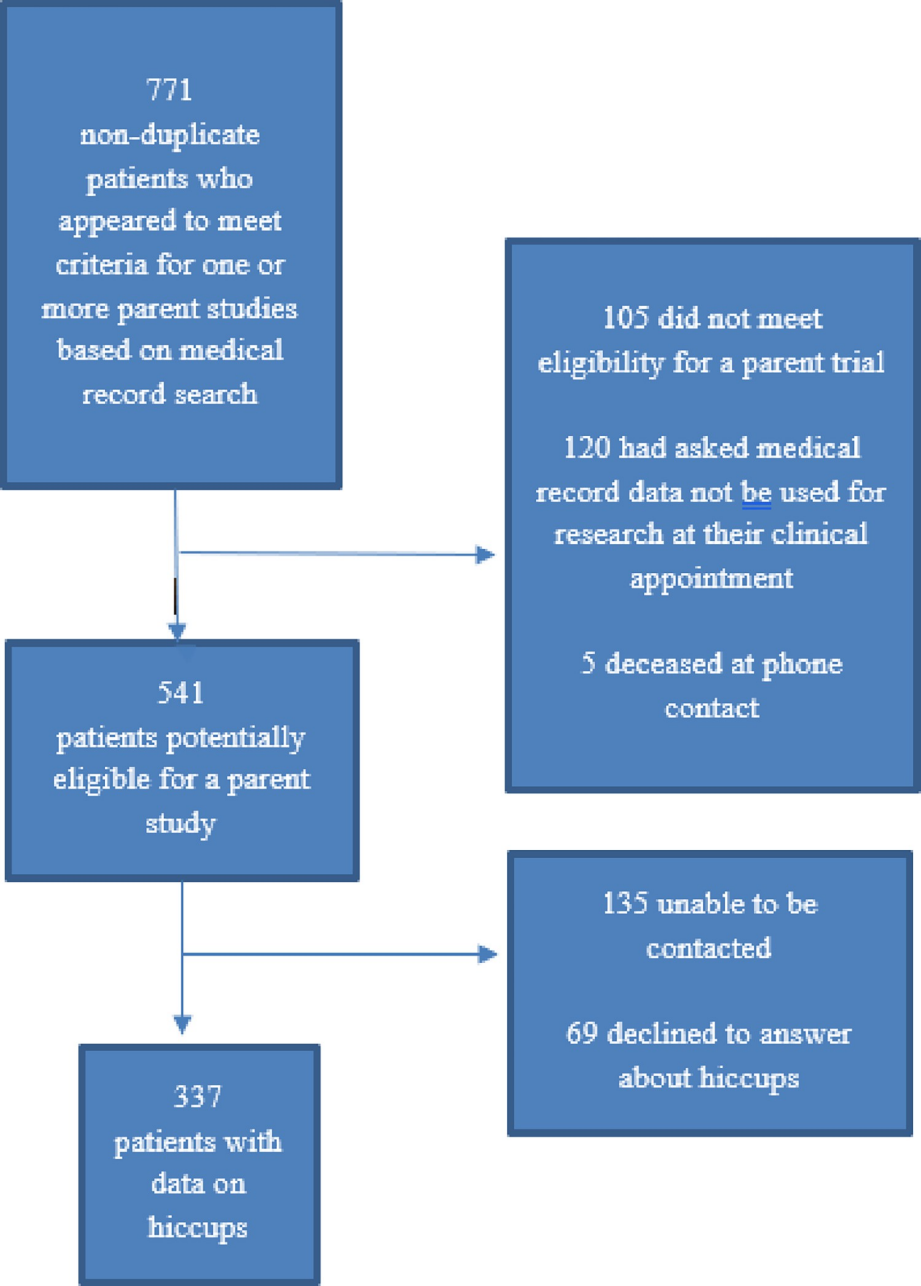
This consort diagram shows how the final patient sample was derived.

For each respective parent study, recruitment had occurred from September 20, 2021 through October 18, 2021 and from December 3, 2021 through February 21, 2022. A total of 541 patients, 337 of whom had been successfully contacted and who responded to the eligibility statement/question on hiccups, are the focus of this report (Fig 1).

Demographics for patients with data on hiccups appear in Table 1.

**Table 1:** Demographics and other baseline factors; n = 337.

	PATIENTS with HICCUPS	PATIENTS with NO HICCUPS
	n = 95	n = 242
	(%)	(%)
Mean age in years	61 (13)	63 (12)
(standard deviation)		
Sex		
Male	68 (72)	130 (54)
Female	27 (28)	112 (46)
Mean height in centimeters	173 (9)	170 (10)
(standard deviation)		
Cancer type		
Gastrointestinal	52 (55)	155 (64)
Head and neck	13 (14)	28 (12)
Other*	30 (31)	59 (24)
Chemotherapy		
Cisplatin, single agent	16 (17)	30 (12)
Cisplatin + other chemotherapy	26 (27)	77 (32)
Oxaliplatin + other chemotherapy	53 (56)	135 (56)
Dexamethasone prescribed?		
Yes	94 (99)	240 (99)
No	1 (1)	2 (1)
Aprepitant or Fosaprepitant prescribed?		
Yes	71 (75)	138 (57)
No (or unknown)	24 (25)	104 (43)

*Other consisted primarily of lung, bladder, cervical, and testicular cancer.

Of note, 204 patients had a complete absence of data on hiccups. A sensitivity analysis that focused on these patients found no statistically significant demographic differences between patients with and without data on hiccups (S1 Table).

### Primary endpoint and variables associated with hiccups

Ninety-five of 337 patients (28%; 95% CI: 23%, 33%) reported hiccups.

In univariable analyses, male gender, increased height, and concomitant aprepitant/fosaprepitant were associated with hiccups (Table 2). In multivariable analyses, these statistically significant associations persisted with the exception of height (Table 2).

**Table 2:** Logistic regression models for hiccups.

	UNVARIABLE ANALYSES		MULTIVARIABLE ANALYSES	
	odds ratio (95% confidence intervals)	p-value	odds ratio (95% confidence intervals)	p-value
Age				
1 year increase (referent: younger)	0.99	0.26	--	--
	(0.97, 1.01)			
Sex				
Female (referent)	2.17	0.002	2.32	0.02
Male	(1.30, 3.62)		(1.12, 4.81)	
Height				
1-cm increase (referent: shorter)	1.03	0.02	1.00	0.97
	(1.00, 1.06)		(0.96, 1.04)	
Chemotherapy				
Cisplatin-based (referent)	1.00	0.99	--	--
Oxaliplatin-based	(0.62, 1.61)			
Aprepitant/Fosaprepitant				
no (referent)	2.23	0.002	2.37	0.001
yes	(1.31, 3.78)		(1.39, 4.07)	

## Discussion

To our knowledge, this study is the first to attempt to acquire consecutive, patient-reported data on hiccups in chemotherapy-treated cancer patients, who had received oxaliplatin- or cisplatin-based chemotherapy. We found that 28% had hiccups. This percentage is in keeping with what others have previously reported with chemotherapy-induced hiccups, underscores that the development of hiccups is not a rare event, but, for the first time, provides important patient-reported data on hiccups [3, 4]. The finding that over one-quarter of patients reported hiccups points to a need for healthcare providers to ask patients about hiccups and to proactively offer palliative options to help with this chemotherapy-induced adverse event.

Importantly, the response rate in this study was slightly over 60%. Other studies have described lower response rates of 10–60% [10]. Thus, the findings reported on the incidence of hiccups in these chemotherapy-treated patients are important not only because they provide patient-reported data but also because they are robust and reflective of a high response rate.

In keeping with previous studies, we observed that in both univariable and multivariable analyses, male gender and concomitant use of aprepitant/fosaprepitant were associated with hiccups [4, 7]. These variables of male gender and use of aprepitant/fosaprepitant should perhaps trigger healthcare providers to make a special point of querying men and those prescribed aprepitant/fosaprepitant about hiccups. It is unclear how male gender and aprepitant/fosaprepitant cause hiccups, as the published literature offers little explanation. One could perhaps speculate that the effects of androgen on muscle and perhaps similar effects of aprepitant/fosaprepitant on muscle or nerves might account for this observed association with hiccups. Clearly, focused research on etiology is needed. Also, of note, in our univariable analyses, increased height was associated with hiccups; but, in multivariable analyses, this association lost its statistical significance. Although others have described increased height as a risk factor for hiccups, our analyses seem to suggest that height is not independently associated with hiccups and that it is perhaps a reflection of the fact that men, who appear strongly predisposed to developing hiccups, tend to be taller [4].

The current study has limitations. First, although we had attempted to recruit consecutively, we were unable to do so in the strictest sense, as our attempts to contact 204 patients yielded absolutely no data on hiccups. Importantly, sensitivity analyses showed no statistically significant demographic differences between patients with and without data on hiccups, an observation that suggests this absence of data is unlikely to inject bias into the findings reported here. Nonetheless, despite our best efforts, we cannot claim that the current study includes a comprehensive group of consecutively recruited patients. Second, nearly all patients had been administered concomitant dexamethasone. Because dexamethasone can cause hiccups on its own right in an estimated 1 in 10 patients, its widespread use in the current study makes it impossible to discern to what extent this drug contributed to the development of hiccups [5, 11]. Clearly, the incidence of hiccups reported here is higher than that reported with dexamethasone alone; and clearly, previous studies indicate that chemotherapy and dexamethasone independently induce hiccups [5, 11]. However, the data reported here leave us unable to parse out the contribution of dexamethasone to this hiccup percentage of 28%. Third, the current study reported only the incidence of hiccups and did not attempt to assess secondary symptoms, such as sleep deprivation, fatigue, pain in the chest and abdomen, and poor oral intake. The design of this study, which relied on previously gathered data, precludes the acquisition of other such important data. It now seems of seminal importance to probe further and undertake research to understand the severity and implications of hiccups among oxaliplatin- and cisplatin-treated patients with cancer. Fourth, as a survey study, the current study is unable to probe into mechanisms of how the chemotherapy agents oxaliplatin and cisplatin cause hiccups. Again, we can only speculate, but both these agents have effects on peripheral nerves, which may somehow predisposition to triggering spasms of the diaphragm with resulting hiccups. Further mechanistic research is indicated.

In summary, the current study found that over one quarter of cancer patients who are prescribed the chemotherapy drugs cisplatin and oxaliplatin–typically in conjunction with dexamethasone–develop hiccups. Future research on hiccups in patients with cancer can potentially use these data as a benchmark for the conduct of much needed research on this topic, including mechanistic research.

## Supporting information

S1 Table Demographics and other baseline factors; n = 541. (DOCX)

S1 File Primary data. (XLSX)
