# Supplementary material for: What percentage of patients with cancer develop hiccups with oxaliplatin- or cisplatin-based chemotherapy? a compilation of patient-reported outcomes
Source: PLoS One. 2023 Jan 27;18(1):e0280947. doi: 10.1371/journal.pone.0280947 (PMC9882886; doi:10.1371/journal.pone.0280947)
Supplement: S1 Table — (DOCX) [file pone.0280947.s001.docx]

SUPPLEMENTARY TABLE 1: Demographics and Other Baseline Factors; n=541

|  | PATIENTS  with HICCUPS DATA (regardless of whether hiccups were reported)  n=337  (%) | PATIENTS with NO HICCUPS DATA  n= 204  (%) | p-value |
| --- | --- | --- | --- |
| Mean age in years  (standard deviation) | 63 (13) | 62 (13) | 0.65 |
| Sex  Male  Female | 198(59)  115 (41) | 130 (64)  89 (36) | 0.58 |
| Mean height in centimeters  (standard deviation) | 171 (10) | 171 (9) | 0.96 |
| Cancer type  Gastrointestinal  Head and neck  Other | 207 (61)  41 (12)  89 (27) | 120 (59)  38 (19)  46 (22) | 0.34 |
| Chemotherapy  Cisplatin, single agent  Cisplatin + other chemotherapy  Oxaliplatin + other chemotherapy | 46 (14)  103 (31)  188 (55) | 34 (17)  71 (35)  99 (48) | 0.25 |
| Dexamethasone prescribed?  Yes  No | 334 (99)  3 (1) | 203 (99)  2 (1) | 0.59 |
| Aprepitant or Fosaprepitant prescribed?  Yes  No (or unknown) | 209 (62)  128 (38) | 135 (66)  60 (34) | 0.33 |
